# EST-PAC a web package for EST annotation and protein sequence prediction

**DOI:** 10.1186/1751-0473-1-2

**Published:** 2006-10-12

**Authors:** Yvan Strahm, David Powell, Christophe Lefèvre

**Affiliations:** 1 Victorian Bioinformatics Consortium, Monash University, Clayton Vic 3800, Australia; 2 Department of Zoology, the University of Melbourne, Melbourne Vic 3010, Australia

## Abstract

With the decreasing cost of DNA sequencing technology and the vast diversity of biological resources, researchers increasingly face the basic challenge of annotating a larger number of expressed sequences tags (EST) from a variety of species. This typically consists of a series of repetitive tasks, which should be automated and easy to use. The results of these annotation tasks need to be stored and organized in a consistent way. All these operations should be self-installing, platform independent, easy to customize and amenable to using distributed bioinformatics resources available on the Internet.

In order to address these issues, we present EST-PAC a web oriented multi-platform software package for expressed sequences tag (EST) annotation. EST-PAC provides a solution for the administration of EST and protein sequence annotations accessible through a web interface. Three aspects of EST annotation are automated: 1) searching local or remote biological databases for sequence similarities using Blast services, 2) predicting protein coding sequence from EST data and, 3) annotating predicted protein sequences with functional domain predictions. In practice, EST-PAC integrates the BLASTALL suite, EST-Scan2 and HMMER in a relational database system accessible through a simple web interface. EST-PAC also takes advantage of the relational database to allow consistent storage, powerful queries of results and, management of the annotation process. The system allows users to customize annotation strategies and provides an open-source data-management environment for research and education in bioinformatics.

## Findings

An expressed sequences tag (EST) is the result of sequencing a portion of a cDNA clone derived from an mRNA [1]. EST sequencing is especially useful for gene discovery in species lacking a draft genome sequence. Used in conjunction with genomic sequencing, ESTs have been used to characterize gene expression products, define intron-exon boundaries, find genes or gene locations [2,3], and analyze splice variation and polymorphism [4]. In many instances EST annotation allows the identification or functional prediction of cloned inserts. These clones can be used subsequently in the laboratory for the experimental characterization of bioactivity. The annotation of EST libraries requires a number of repetitive tasks (see [5] for a review) that are easily automated: database searches, translations into peptides and, functional annotation of translation products with probabilistic model searches such as the protein family database (Pfam) [6]. Efforts in the open source and in the academic community have been made to provide the scientific community with on line services, examples of which are PipeOnline, [7], EST-PAGE [8], or complete packages such as ESTannotator [9], ESTAP [10], PartiGene [11], and Prot4EST [12]. However, these packages often have restrictive system dependencies, do not always allow extensive data mining and, may not always be available for download and customization. Furthermore, few packages allow real sequence management where users can decide to build, store and use complex filters for sequence similarity searches with criteria based on previous results. This facility offers more flexibility for the annotation process, updating and the optimal use of often limited computational resources. Here, we propose a flexible interface and a PHP Hypertext Preprocessor programming language framework for annotation data mining. The management and updating of sequence annotations is facilitated using EST-PAC and the source code is accessible for customization of the web interface.

The core of EST-PAC consists of an open source relational database management system that uses Structured Query Language (MySQL) and a number of PHP programs. EST-PAC uses open source software; MySQL 4 or 5 [13] for database management and, PHP 4 or PHP 5 [14] for web development. PHP allows the storage and management of ESTs using web pages. User login is available for visualization and query only or, with additional privileges to run annotation tools. Sequences in FASTA format are loaded into the database through a web interface and annotation tasks can be requested. A set of continuously running programs checks the database and extracts sequence to be processed using the BLASTALL suite [15], ESTScan2 [16,17] or, HMMER [18] against the Pfam database [19]. The coding content of the EST can be evaluated with the Hidden Markov Model approach of ESTScan2 and the predicted translation products can then be compared against protein sequence databases. A report can be obtained from a web query page. As all results are stored in a relational database, users are able to query on every value returned by the annotation process. An interface is also available to assist the construction and storage of database queries. In addition to the public databases which can be downloaded and installed locally or accessed through web based blast services such as NCBI, users have the possibility to create their own databases from EST-PAC in order to make more precise and relevant comparisons. We have tried to restrict the programming in EST-PAC to the PHP language. However, Perl 5.8.1 was used to integrate ESTScan2. The usage of Perl is limited to this and a full installation of Perl or BioPerl [20] is not necessary. System specific configuration of EST-PAC has been kept to a minimum. However, some indispensable set-up is needed. First, MySQL should be running and MySQL administrator login and password are required during installation. Second the configuration file (config.inc.php) should be edited to reflect the environment where the package is installed. The user must indicate where in the operating system the package and auxiliary programs are located as well as the name of the database to be created. It is also possible to create multiple databases by specifying database names, usernames, and passwords. These parameters will be passed automatically at the time of database creation. The BLASTALL programs can be used either locally or remotely. With the remote option, blast jobs will be sent to the blast server at NCBI [21] in compliance with NCBI remote access policy. Access to other resources can also be implemented using the EST-PAC framework.

EST-PAC is distributed in a comprehensive PHP package for the integration of all the third party bioinformatics annotation pipeline program components. Installation of the auxiliary components (NCBI tool suite version 2.2.10, HMMER version 2.3.2 ESTSCAN version 2.0b, MySQL version 5, and PHP) is also necessary. EST-PAC scripts can be downloaded [22]. Installation and configuration instructions are available here for MacOSX, Windows or Linux systems. In addition, precompiled version of ESTScan2 for MacOSX, Linux Fedora Core 3, Centos 4 and Windows are also available at this URL.

EST-PAC is a software package for the annotation of EST data particularly geared towards laboratories with limited bioinformatics resources and expertise. It provides a basic package to install a database system for the management of a complete suite of third party annotation software components, integrated into a simple and powerful web interface. Mainly built in PHP, the source code is easily accessible to developers for customization and evolution. EST-PAC does not currently address directly the assembly of EST sequences. It is however possible to assemble EST data independently and straightforward to annotate the contig sequences obtained after the assembly process. Additional support for EST assembly data storage and visualization is in development. Furthermore, EST-PAC already provides a workbench to cluster ESTs onto reference sequence data sets when available, for example, from public genome annotation data. Finally, usage of EST-PAC is not restricted to EST sequences and any type of nucleotide or protein sequences can be loaded for the management of sequence analysis results. This allows the compilation, storage and management of a diversity of customized sequence databases for the analysis of EST or other sequence libraries. In conclusion, EST-PAC provides an open framework for rapid prototyping of data mining and on-line visualization of sequence data, presenting an expandable data-management environment for research and education in bioinformatics.

## Availability

Project name: EST-PAC.

Project home page: .

Operating system(s): multiplatform; Programming language: PHP 4 or 5;

Other requirements: mySQL, BLASTALL suite, ESTScan2 and HMMER.

A web page with a fully functional instance of EST-PAC has been set up for demonstration purpose. Software download and installation instructions are also available under GPL.

## Competing interests

The author(s) declare that they have no competing interests.

## Authors' contributions

YS was involved in the conceptualisation, development and testing of the package. DP was involved in the programming of the core functionality of EST-PAC. CL supervised the work, implemented the graphic components and, prepared the manuscript.

**Figure 1:**
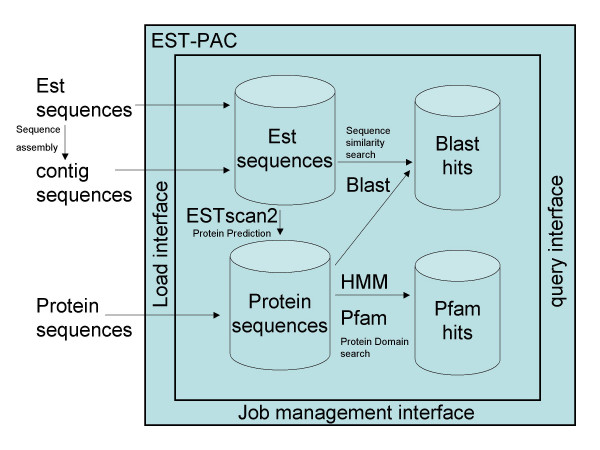
**Workflow and interfaces available in EST-PAC**. EST-PAC provides a web interface for the management, storage and querying of sequences and results from annotation tools such as BLASTALL, EST-Scan2 and HMMER.
